# Monobutyrin can alleviate hepatic lipid dysmetabolism and improve liver mitochondrial ultrastructure and autophagy in high-fat diet mice

**DOI:** 10.1038/s41538-025-00524-6

**Published:** 2025-07-29

**Authors:** Yuqing Zhang, Xiaoteng Li, Haidong Wang, Kexin Zhang, Ji Qiu, Minyao Zhou, Minqi Wang

**Affiliations:** Key Laboratory of Molecular Animal Nutrition, Ministry of Education, College of Animal Sciences, Zhejiang University, Hangzhou, PR China

**Keywords:** Diseases, Risk factors

## Abstract

The incidence of non-alcoholic fatty liver disease has proportionally escalated alongside the global epidemic of obesity. Monobutyrin (MB), a food additive found in butter and cod liver oil, possesses lipid-regulating properties. This study aimed to explore the alleviating effect of MB on liver oxidative injury and lipid metabolism in obese mice induced by a high-fat diet (HFD). The results showed that MB administration (1 or 2 g/kg body weight (BW)) for 8 weeks significantly reduced body weight, improved hepatic lipid metabolism via activation of the peroxisome proliferator-activated receptor α (PPARα) signaling pathway, and stabilized liver mitochondrial ultrastructure to alleviate oxidative liver injury by triggering mitochondrial autophagy through regulation of microtubule-associated protein 1A/1B-light chain 3 (LC3) and ubiquitin-binding protein (P62) in mice. Moreover, MB might increase the abundance of beneficial bacteria, promote short-chain fatty acid levels, and alleviate high-fat induced obesity via the gut-liver axis. These findings provide a novel insight into MB as an intervention strategy for hepatic metabolic disorders.

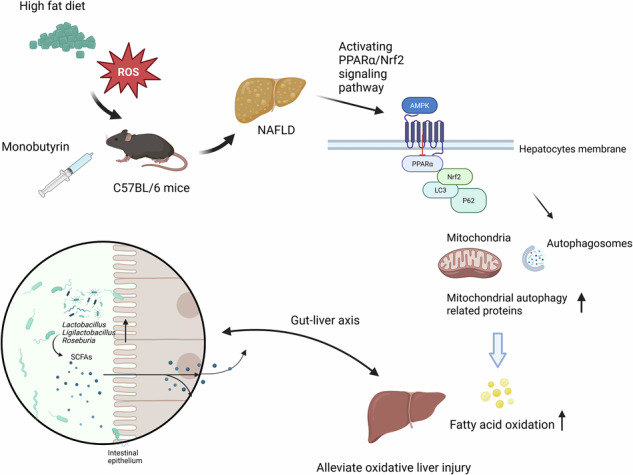

## Introduction

In recent years, the high prevalence of non-alcoholic fatty liver disease (NAFLD) has been recognized as a major health issue of global concern^1^. Excessive accumulation of lipids in the liver leads to oxidative stress, inflammation and mitochondrial dysfunction in hepatocytes^2^. When mitochondrial function is impaired, fatty acids in the liver cannot be completely oxidized, resulting in excessive lipid accumulation, which will continue to aggravate NAFLD^3^. Recent studies have reported that impaired mitophagy is involved in the pathogenesis of NAFLD. Cells maintain mitochondrial function and protein folding by removing damaged mitochondria through mitophagy and activating the AMPK/mTOR autophagy signaling pathway^4^. Activated AMPK can reduce hepatic gluconeogenesis and regulate lipid homeostasis in hepatocytes by activating PPARα^5^.

Butyric acid can significantly reduce plasma total triglycerides and cholesterol by increasing fatty acid oxidation, thereby alleviating diet-induced obesity in mice^6^. In addition, it has been shown to directly inhibit lipid transport and may be involved in cholesterol metabolism^7,8^. However, the use of free butyric acid is limited due to its unpleasant odor and short half-life^9^. Monobutyrin (MB) is classified as a food additive and is naturally present in butter and cod liver oil. Interestingly, MB was first discovered as a novel angiogenic factor secreted during the differentiation of precursor adipocytes. It promotes the formation of microvessels, and its biosynthesis appears to be directly associated with fat breakdown^10^. Notably, synthetic MB is the product formed by esterifying butyric acid with glycerol and is a modified butyrate that overcomes the disadvantages of butyric acid, such as its irritating odor and susceptibility to rancidity^11^. It has been found that synthetic MB exhibits similar efficacy to endogenous MB. It shows low molecular polarity, low affinity for lipase, a stable and non-volatile structure, and good water solubility^12^. Moreover, MB is easily absorbed via intestinal epithelial cells^13^. Westergaard et al. suggested that MB, as an analog of glycerol, can be metabolized through glycerol kinase (GK) and glycerol-3-phosphate dehydrogenase (GDH), thereby inhibiting glycerol uptake into liver cells and reducing cellular lipid deposition^14^. A recent study revealed that MB treatment could decrease the total triglyceride (TG) and cholesterol (TC) levels in the hepatic tissues of HFD-induced rats^15^. Furthermore, butyrate increased the relative abundance of probiotics, particularly *Lactobacillus*, and modulated obesity-associated gut microbiota composition, thereby attenuating fat accumulation^16,17^.

To date, how MB affects lipid metabolism and the underlying mechanisms has not been elucidated. Therefore, the aim of the present study was to investigate the effects and mechanisms of MB supplementation on hepatic lipid metabolism and oxidative stress relief in a mouse model of HFD-induced obesity. The obtained results provide theoretical evidence for developing a strategic approach to prevent and treat hepatic metabolism-associated disorders, as well as for the potential application of MB in obesity treatment.

## Results

### Monobutyrin alleviated weight gain induced by HFD

**Fig. 1:**
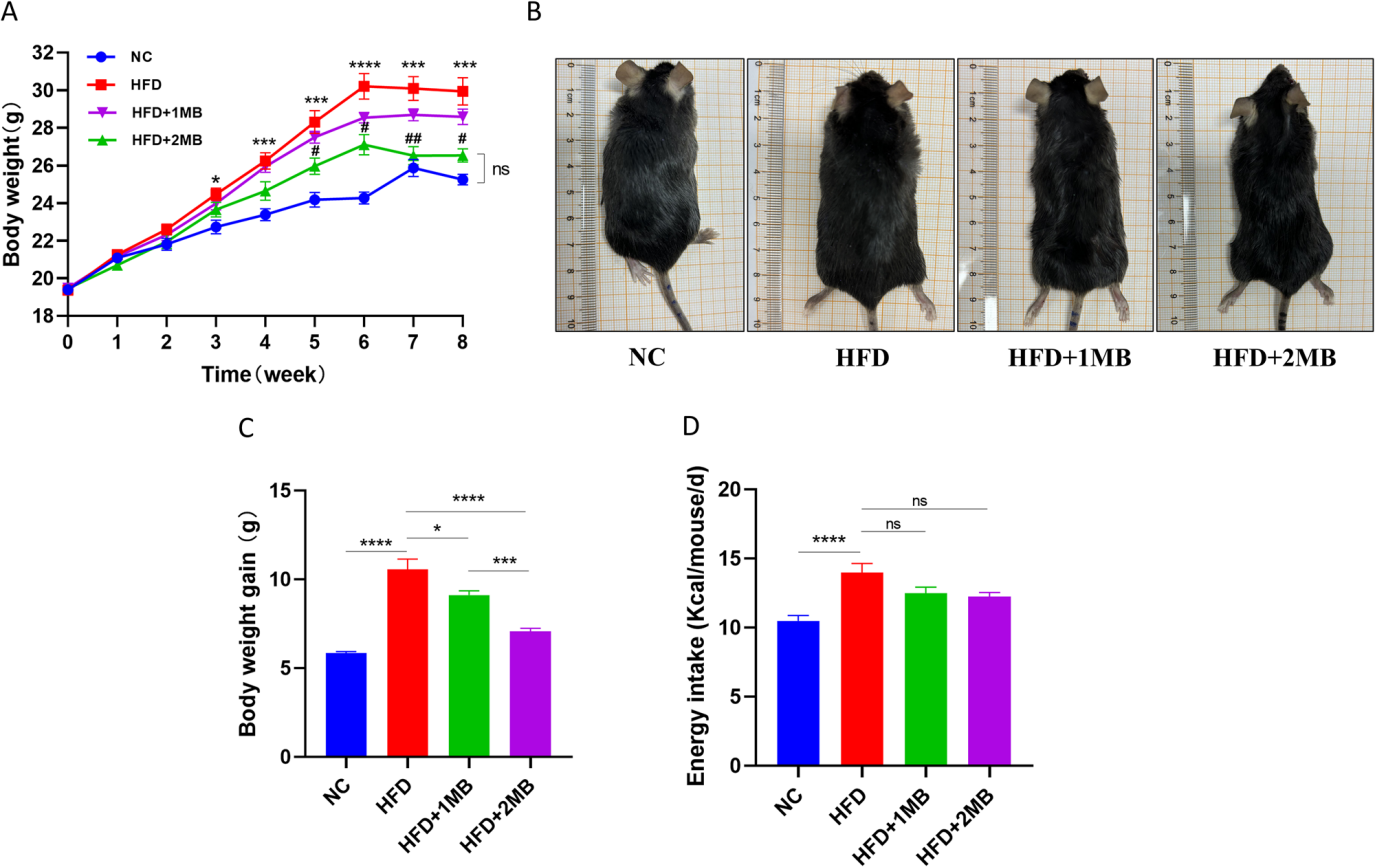
Effect of MB on body weight and food intake of mice. NC mice administered general diet and gavaged with PBS, HFD mice administered high-fat diet and gavaged with PBS, HFD + 1MB mice administered high-fat diet and gavaged with 1 g/kg BW MB; HFD + 2MB, mice administered high-fat diet and gavaged with 2 g/kg BW MB. **A** Changes in body weight during the experiment; * *P* < 0.05, ** *P* < 0.01, *** *P* < 0.001, and **** *p* < 0.0001 were used to compare NC and HFD. ^#^ *P* < 0.05 and ^##^ *P* < 0.01 were used to compare HFD and HFD + 2MB. ^ns^ *p* > 0.05 was used to compare NC and HFD + 2MB. **B** Figures of mice body size at the end of the experiment; **C** Weight gain during the administration period; **D** Energy intake. All data were represented as mean ± SEM, n = 10. The significant difference was analyzed by one-way ANOVA, * *p* < 0.05, ** *p* < 0.01,*** *p* < 0.001,**** *p* < 0.0001, ^ns^ *p* > 0.05.

There was no significant difference in the body weight of the mice at the beginning of the test (*P* > 0.05). As expected, HFD induced significant gains in body weight after the third week. Figure 1 showed that the weight gain of the HFD + 2MB group was considerably reduced compared to that of the HFD group during the MB intervention period after the 5th week (*P* < 0.05), and the HFD + 2MB group was the closest to the NC group for 8 weeks (Fig. 1A-C). Meanwhile, the average daily intake of mice was generally not significantly different among the groups fed high-fat diets, suggesting that the effect of MB on alleviating obesity was not related to food intake (Fig. 1D). The above results indicated that the high-fat diet could significantly induce obesity in mice, and MB had a certain alleviation effect on obesity induced by the HFD diet, while the effect of high-dose MB intervention was more prominent.

### MB improved liver lipid accumulation induced by HFD

**Fig. 2:**
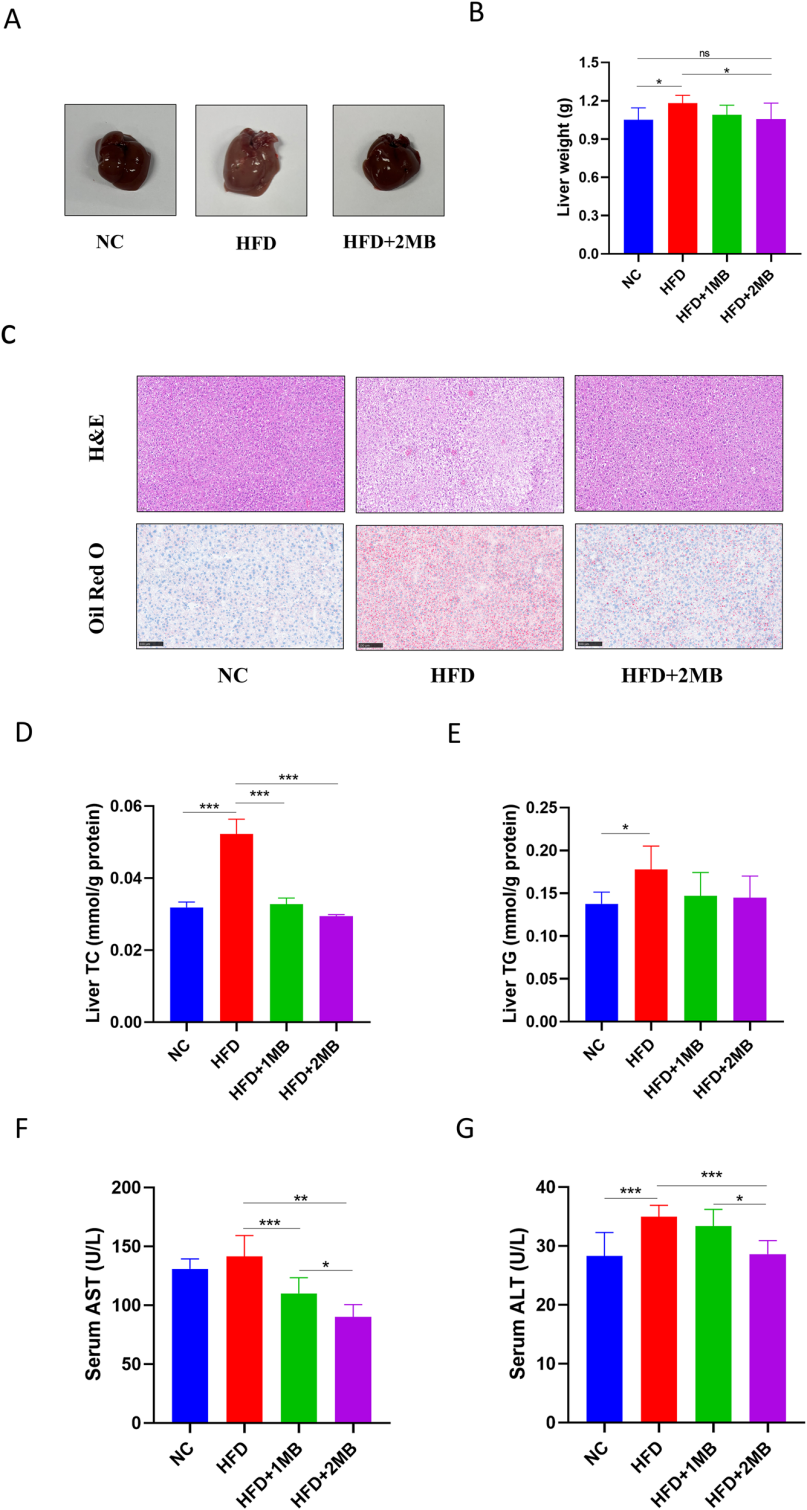
Effect of MB on lipid deposition and morphology changes in mice liver. **A** Representative images of the external morphology; **B** Liver weight; **C** H&E staining (scale bar: 50 μm, magnification: 20×) and Oil Red O (scale bar: 100 μm, magnification: 20×) staining for light microscopy analysis of liver tissues of mice; **D** Liver TC level; **E** Liver TG level; **F** Serum AST level; **G** Serum ALT level. All data were represented as mean ± SEM, n = 8. The significant difference was analyzed by one-way ANOVA, * *p* < 0.05, ** *p* < 0.01, and *** *p* < 0.001, ^ns^*p* > 0.05.

Figure 2A-C showed that the liver weight of mice in the HFD group was extremely significantly increased from that of the NC group (*P* < 0.05) with a yellowish and larger liver. In comparison with the model group, the liver weight of MB-treated mice at higher doses was significantly reduced (*P* < 0.05). The results of H&E and Oil Red O staining revealed neatly arranged hepatocytes with normal hepatic architecture and clearly defined hepatic cords in the NC group. In contrast, liver sections of the HFD group showed extensive intracellular vacuolization, abundant lipid vacuoles and red-stained lipid droplets. Notably, the HFD + 2MB group had fewer fat vacuoles and red lipid droplets compared to the HFD group, suggesting that MB effectively relieved lipid accumulation in the liver of mice by improving histological alterations.

To further evaluate the effect of MB on liver lipid metabolism, serum biochemical parameters related to lipid metabolism were measured. The HFD-fed mice significantly elevated hepatic TC and TG levels, as well as increased serum ALT activity, compared with the NC group (*P* < 0.05, Fig. 2D-G). However, MB intervention significantly attenuated these adverse changes in the HFD-fed mice. The HFD-fed mice treated with MB showed significantly lower hepatic TC as well as serum AST and ALT levels compared with the HFD group (*P* < 0.05).

### MB alleviated HFD-induced oxidative stress in obese mice

**Fig. 3:**
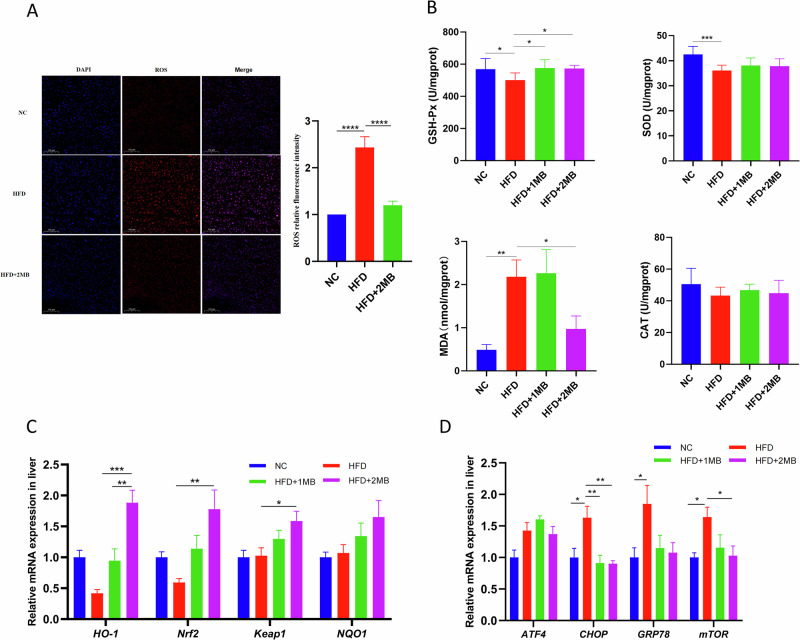
Effects of MB on oxidative stress state of mice. **A** The representative fluorescence image and relative fluorescence intensity of ROS (red) in the liver (n = 3); **B** The hepatic redox homeostasis indexes (n = 8); **C** Expression of genes related to antioxidant capacity in the Nrf2 signaling pathway; **D** Expression of genes related to endoplasmic reticulum stress. Data are presented as mean ± SEM, n = 6. * *p* < 0.05, ** *p* < 0.01,*** *p* < 0.001.

As shown in Fig. 3, we employed immunofluorescence to assess ROS levels and measured antioxidant enzyme activities. The results revealed that ROS fluorescence intensity was increased after high-fat diets and attenuated after the addition of high-dose MB (Fig. 3A). Furthermore, in comparison to the NC group, the HFD-fed mice exhibited a significant decrease in GSH-Px, and SOD activity, and an evident increase in MDA content (*P* < 0.05, Fig. 3B). However, the HFD + 2MB group significantly attenuated the GSH-Px activities, as well as MDA content, in line with the result of ROS fluorescence intensity (*P* < 0.05). To explore the mechanism by which MB attenuates oxidative stress, the expression levels of antioxidant factors and endoplasmic reticulum (ER) stress in the liver were assessed (Fig. 3C, D). Mice exposed to the HFD showed higher mRNA expression levels of endoplasmic reticulum stress markers, including *CHOP*, *GRP78*, and *mTOR*. Compared with the HFD group, the HFD + 2MB group showed significantly upregulated expression of antioxidant factors *HO-1*, *Nrf2*, and *Keap1*, as well as significantly downregulated expression of ER stress markers *CHOP* and *mTOR* (*P* < 0.05). The comprehensive results indicated that MB could alleviate HFD-induced oxidative stress and liver endoplasmic reticulum stress by activating the Nrf2 signaling pathway.

### MB stabilized liver ultrastructure and removed damaged mitochondria in mice

**Fig. 4:**
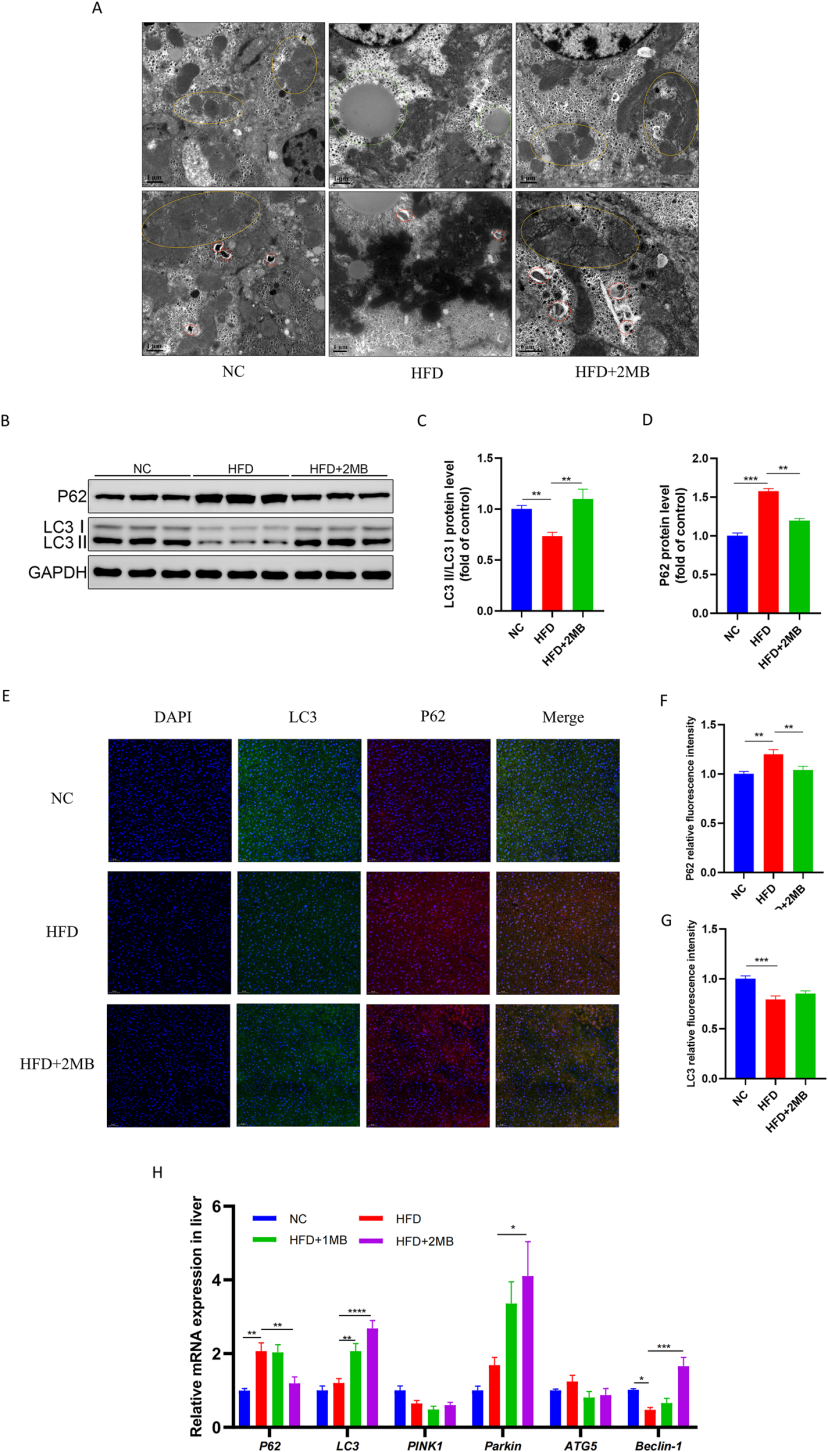
Effects of MB on ultrastructure and mitochondrial autophagy of the liver. **A** Representative TEM images. The areas in the yellow circles show mitochondria; the areas in the red circles show autophagosome; the areas in the green circles show lipid droplets (scale bar: 1 μm, magnification: 15,000–20,000×, n = 3). **B** Western blotting images of LC3 and p62 (n = 3); **C** Relative protein expression of LC3; **D** Relative protein expression of P62; **E** Representative LC3 (green) and p62 (red) immunofluorescence staining images in hepatic tissue. Nuclei were stained with DAPI (blue) (scale bar: 50 μm, magnification: 200×, n = 3); **F** P62 relative fluorescence intensity; **G** LC3 relative fluorescence intensity; **H** Expression of genes related to liver mitochondrial autophagy. Data are presented as mean ± SEM, n = 6. * *p* < 0.05, ** *p* < 0.01,*** *p* < 0.001, **** *p* < 0.0001.

The ultrastructure of the mouse liver was observed by transmission electron microscope. As shown in Fig. 4A, the number of normal mitochondria was abundant in the NC group, the mitochondrial double membrane structure was clear, and there were autophagosomes. HFD intake induced swollen mitochondria and a significant reduction in mitochondrial cristae. The mitochondrial cristae structure was unclear, and some lipid droplets were also seen in the cytoplasm. However, these adverse effects were significantly attenuated by the intake of MB; the mitochondrial structure was complete, and more autophagosomes were formed. These findings suggest that MB may stabilize liver ultrastructure by promoting autophagy and eliminating damaged mitochondria.

### MB triggered mitochondrial autophagy in obese mice

We further conducted the western blot analysis of autophagy-related proteins P62 and LC3, as well as immunofluorescence staining to visualize autophagy expression and distribution in the liver. The results of WB showed an evident inhibition of p62 expression in the HFD + 2MB group (*p* < 0.05). The LC3 is a key marker of autophagy initiation, converting from type I (LC3-I) to type II (LC3-II). In the HFD group, the LC3-II/LC3-I ratio was markedly reduced, while MB effectively mitigated this process (*p* < 0.05, Fig. 4B–D). Furthermore, the immunofluorescence analysis showed a weak LC3 fluorescence intensity and a strong P62 fluorescence intensity in the HFD groups. MB treatment restored autophagy activity, as evidenced by increased LC3 and decreased P62 fluorescence intensity (*p* < 0.05, Fig. 4E–G). These results illustrated that MB effectively remitted the inhibition of autophagy generated by HFD.

Moreover, the expression of autophagy-related genes was evaluated in Fig. 4H. The results showed that *Beclin-1* mRNA levels were significantly decreased, while *P62* mRNA levels were significantly increased in the HFD group. Furthermore, compared to the HFD group, the HFD + 2MB group showed upregulated expression of *LC3*, *Parkin*, and *Beclin-1*, and downregulated expression of *P62* (*P* < 0.05).

MB administration on hepatic gene expression associated with lipid metabolism

**Fig. 5:**
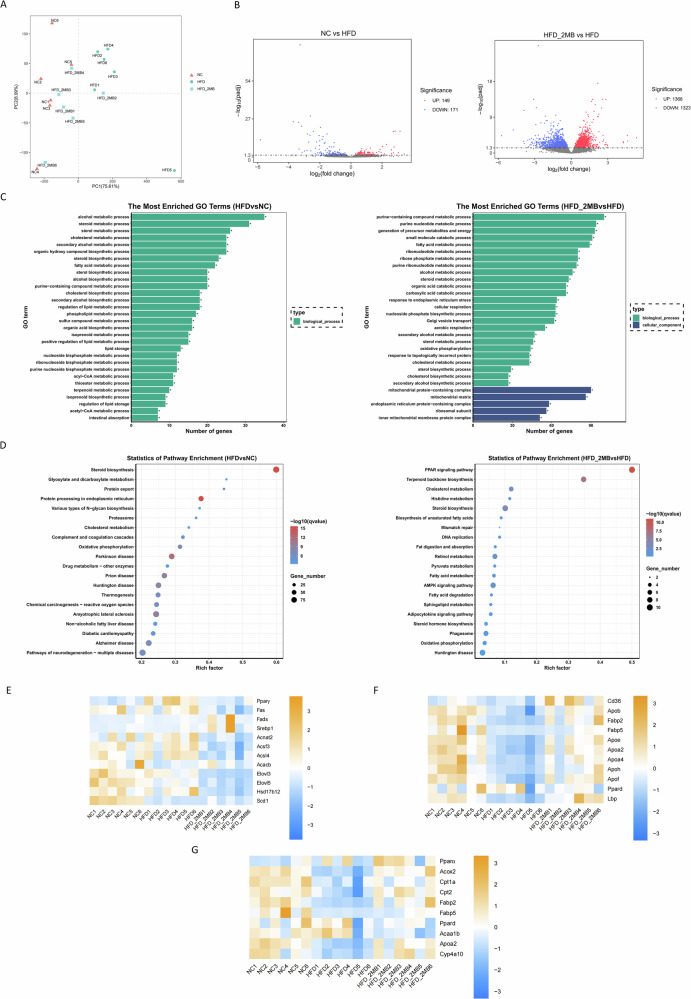
MB regulates hepatic gene expression profiles in mice. **A** PCA score plots; **B** Volcano plot of DGEs. Each dot represents a particular gene, and its corresponding horizontal and vertical values are gene expression changes in the NC and HFD groups or HFD and HFD + 2MB groups, respectively. The red dots mean up-regulated genes expressed significantly, and the blue dots mean down-regulated genes expressed significantly. **C** GO enrichment analysis. The vertical axis represents GO terms and the horizontal axis is the Rich factor. **D** KEGG pathway analysis. **E** Heatmap cluster analysis of DGEs in the fatty acid biosynthesis pathways; **F** Heatmap cluster analysis of DGEs in the lipid transport pathways; **G** Heatmap cluster analysis of DGEs in the PPAR signaling pathways related to fatty acid oxidation. n = 6 mice per group.

**Fig. 6:**
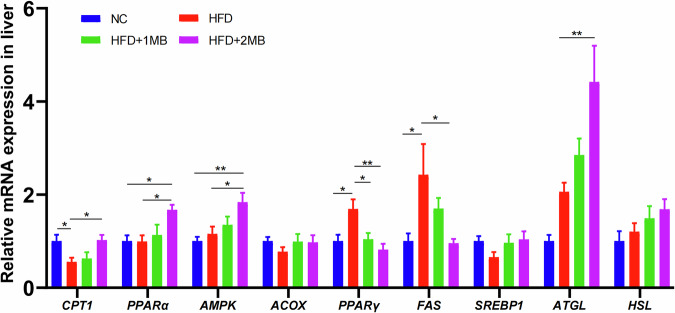
Effect of MB on mRNA expression of genes related to liver lipid metabolism. Relative mRNA expression of *CPT1*, *PPARα*, *AMPK*, *ACOX*, *PPARγ*, *FAS*, *SREBP1*, *ATGL* and *HSL*. Data were represented as mean ± SEM, n = 6. The significant difference was analyzed by one-way ANOVA,* *p* < 0.05, ** *p* < 0.01,*** *p* < 0.001.

Considering that MB intervention could attenuate hepatic lipid deposition caused by HFD, we then investigated the effect of MB on the mRNA levels of hepatic genes associated with lipid metabolism based on RNA sequencing methods. PCA showed that the HFD group was significantly separated from the NC group, while the gene clustering from the HFD + 2MB group was closer to the NC group (*P* < 0.05, Fig. 5A). Compared with NC mice, HFD-induced mice showed 320 differentially expressed genes (DEGs), and MB treatment resulted in 2691 DEGs, of which 1368 were up-regulated and 1323 down-regulated (Fig. 5B). GO and KEGG analyses were performed for the total DEGs in each group. The results showed that the number and the GO category of DEGs in the liver of each group were obviously different. The top 30 GO terms with the most significant enrichment were selected, as shown in Fig. 5C, which demonstrated several enriched biological processes involved in lipid metabolism in DEGs in the HFD group, such as steroid, sterol, and cholesterol metabolism. However, MB mainly altered the genes associated with cellular components such as mitochondrial protein, endoplasmic reticulum protein, and inner mitochondrial membrane protein complex. A KEGG pathway analysis was carried out to obtain more information on the prediction functions of the DEGs. The top 20 KEGG pathways with the most significant enrichment were selected, as shown in Fig. 5D, among which the PPAR signaling pathway, AMPK signaling pathway, and non-alcoholic fatty liver disease pathways were most commonly affected. Further, heatmap cluster analysis of 33 DEGs related to lipid metabolism revealed that, compared with the HFD mice, genes related to fatty acid biosynthetic processes such as peroxisome proliferator-activated receptor γ (*PPARγ*), fatty acid synthase (*Fas*), acyl-coenzyme A amino acid N-acyltransferase 2 (*Acnat2*), acyl-CoA synthetase family member 3 (*Acsf3*), acyl-CoA synthetase long-chain family member 4 (*Acsl4*), fatty acid elongase 3 (*Elovl3*), fatty acid elongase 5 (*Elovl5*) and hydroxysteroid dehydrogenase 12 (*Hsd17b12*) were significantly down-regulated in the MB mice (Fig. 5E). Moreover, genes related to lipid transport such as CD36 molecule (*Cd36*), apolipoprotein B (*Apob*), fatty acid binding protein 2 (*Fabp2*), fatty acid binding protein 5 (*Fabp5*), apolipoprotein E (*Apoe*), apolipoprotein A-II (*Apoa2*), apolipoprotein A-IV (*Apoa4*) and lipopolysaccharide-binding protein (*Lbp*) were significantly down-regulated in HFD mice, while were suppressed by MB treatment (Fig. 5F). It was noteworthy that MB could advance PPAR signaling pathway related to fatty acid oxidation by up-regulating the gene expression levels of peroxisome proliferator-activated receptor α (*PPARα*), acyl-Coenzyme A oxidase 2 (*Acox2*), carnitine palmitoyltransferase 1 (*Cpt1*), carnitine palmitoyltransferase 2 (*Cpt2*) and fatty acid binding protein 2 (*Fabp2*), thus promoting catabolism in cells and regulating lipid degradation in the body. These results suggested that the anti-NAFLD effect of MB was associated with the inhibition of fatty acid synthesis and promotion of β-oxidation in the liver of HFD-fed mice (Fig. 5G). Notably, the reliability of RNA-seq data was verified by qRT-PCR for randomly selected genes (Fig. 6).

### Verification of RNA-Seq results through qRT-PCR in the liver

To prove the correctness of the results obtained from RNA-seq analysis, qRT-PCR was employed to analyze the mRNA expression of nine genes selected randomly. The mRNA expression of the lipogenesis-related gene *PPARγ* and *FAS* was significantly elevated and the adipose differentiation-associated gene *CPT* was significantly decreased with HFD stimulation in comparison to untreated controls (*p* < 0.05, Fig. 6). Interestingly, we found that high-dose MB treatment significantly downregulated the mRNA expression of the lipogenic gene *PPARγ* and *FAS*. However, fatty acid oxidation-related gene *CPT*, *PPARα*, lipolysis-related genes *ATGL*, and the regulator of energy metabolism such as *AMPK* mRNA levels were considerably higher in the HFD + 2MB group than in the HFD group (*p* < 0.05). In brief, the expression trends of the detected genes were highly in conformance with the results obtained by RNA-seq.

### MB changed the diversity and composition of gut microbiota

**Fig. 7:**
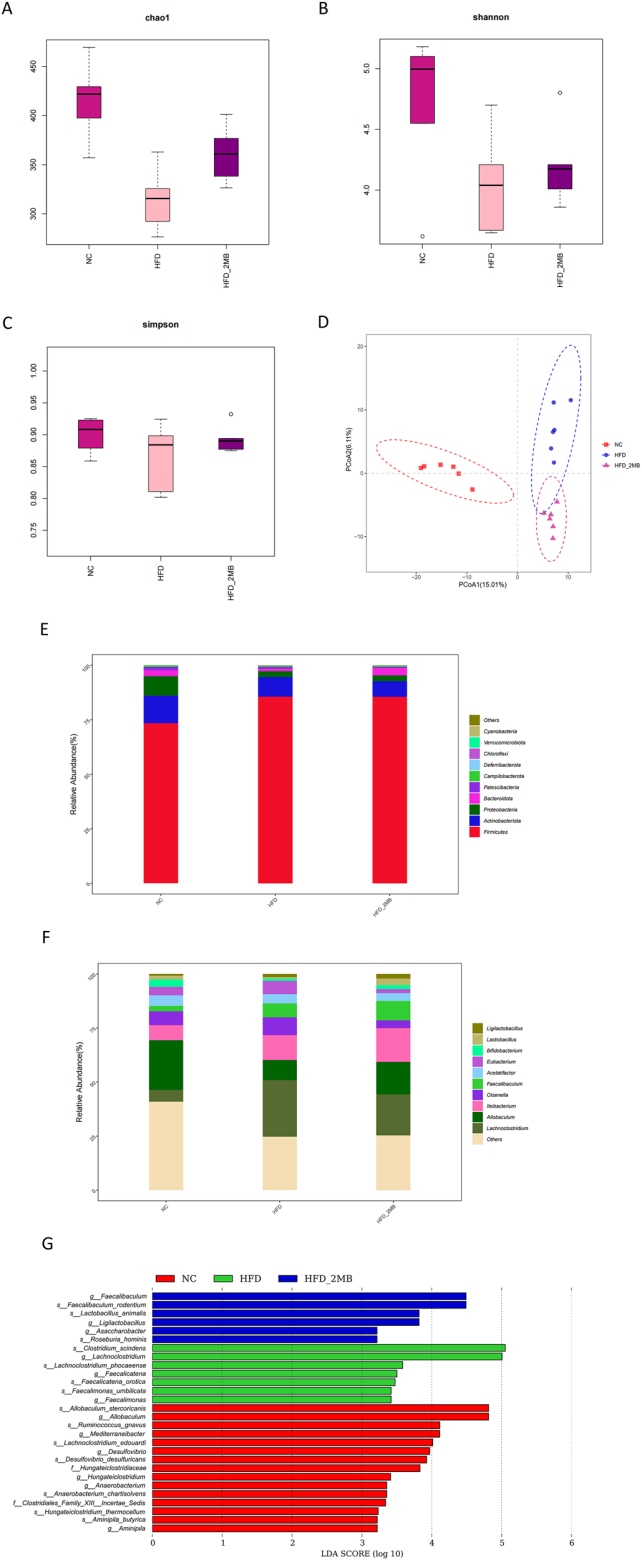
α diversity, β diversity analysis and microbial composition of colon content in mice. The α-diversity was evaluated by **A** the Chao1 index, **B** the Shannon index, and **C** the Simpson index. β-diversity was evaluated via **D** principal coordinates analysis (PCoA) based on the Bray–Curtis distance; **E** Microbial community bar plot at phylum level; **F** Microbial community bar plot at genus level; **G** Lefse (LDA effect size) analysis in which the LDA scores ≥ 3. n = 6 mice per group.

To assess the effects of MB on the composition of intestinal microbiota, the V3–V4 regions of the 16S rRNA gene were sequenced using the high-throughput sequencing technology based on the Illumina MiSeq platform. Alpha diversity encompassed both richness (Chao1 index) and diversity (Shannon and Simpson index) of the gut microbiota. As compared to the NC group, the Chao1 index and Shannon index were substantially lower in the HFD group (*p* < 0.05, Fig. 7A–C, Table 1). However, mice fed a high dose of MB exhibited a significant increase in the chao1 index (*p* < 0.05), suggesting that although a high-fat diet reduced the richness and diversity of the intestinal flora, MB intervention could mitigate this reduction.

**Table 1:** Effects of MB on the a-diversity of colon content in mice

Parameter	NC	HFD	HFD_2MB	SEM	*P*-value
Chao1	416.20^a^	314.79^c^	360.80^b^	12.30	<0.001
Shannon	4.74^a^	4.05^b^	4.20^ab^	0.12	0.043
Simpson	0.90	0.87	0.89	0.01	0.228

^a,b,c^Means that do not share common letters differ significantly (*P* < 0.05).

Then, β-diversity was evaluated via principal coordinates analysis (PCoA) based on the Bray-Curtis distance. It was shown that the NC group exhibited an obvious separation in microbiota structure compared with the HFD and HFD + 2MB groups (*p* < 0.05, Fig. 7D). Meanwhile, there was also a discrepancy in the composition of gut bacteria between the HFD and HFD + 2MB groups, indicating that the gut microbial community in mice fed with the HFD was influenced by MB administration.

At the phylum level, *Firmicutes*, *Proteobacteria*, *Actinobacteriota*, and *Bacteroidota* accounted for more than 90% of the total colonic microbiota. Compared to the NC group, the supplementation of the HFD increased the richness of *Firmicutes* significantly (*p* < 0.05, Fig. 7E, Table 2). At the genus level, the proportions of *Lachnoclostridium* were significantly increased, whereas *Allobaculum* was significantly decreased in mice of the HFD group than those of normal controls. Notably, the alterations were attenuated by MB supplementation. In particular, the richness of *Lachnoclostridium* was significantly reduced by MB in HFD-induced mice (*p* < 0.05, Fig. 7F, Table 3).

**Table 2:** Effects of MB supplementation on fecal microbial composition at phylum level in mice

Parameter(%)	NC	HFD	HFD_2MB	SEM	*P*-value
*Firmicutes*	73.38^b^	85.66^a^	85.60^a^	2.350	0.034
*Proteobacteria*	9.02	2.69	2.72	1.289	0.056
*Actinobacteriota*	12.68	8.99	7.11	2.006	0.550
*Bacteroidota*	2.74	1.14	3.33	0.730	0.483
*Patescibacteria*	1.48	0.81	0.50	0.229	0.207
*Campilobacterota*	0.59	0.55	0.66	0.197	0.980
*Deferribacterota*	0.02	0.04	0.04	0.011	0.629
*Cyanobacteria*	0.06	0.00	0.00	0.017	0.250
*Chloroflexi*	0.02	0.05	0.03	0.008	0.358
*Verrucomicrobiota*	0.01	0.05	0.01	0.008	0.067

^a,b^Means that do not share common letters differ significantly (*P* < 0.05).

**Table 3:** Effects of MB supplementation on fecal microbial composition at the genus level in mice

Parameter(%)	NC	HFD	HFD_2MB	SEM	*P*-value
*Allobaculum*	23.04^a^	9.37^b^	15.08^b^	1.94	0.006
*Ileibacterium*	6.96	11.45	15.62	1.45	0.140
*Olsenella*	6.34	8.25	3.55	1.44	0.434
*Lachnoclostridium*	5.40^b^	26.16^a^	15.98^b^	3.05	0.009
*Acetatifactor*	4.90	4.27	3.70	0.61	0.751
*Eubacterium*	3.89	6.13	1.80	0.51	0.330
*Bifidobacterium*	3.43	1.12	1.94	0.47	0.120
*Faecalibaculum*	2.54	6.43	8.93	1.13	0.056
*Lactobacillus*	1.78	0.67	2.96	0.63	0.351
*Ligilactobacillus*	0.74	1.40	2.12	0.25	0.072

^a,b^Means that do not share common letters differ significantly (*P* < 0.05).

We performed LEfSe analysis with an LDA score ≥ 3 to further explore the biomarker with statistical differences among all groups. Overall, 28 microbial groups were statistically different among the three groups. Especially, the abundance of *Allobaculum*, *Ruminococcus*, and *Aminipila* had a significant impact on the NC group. The predominant species in the HFD group were *Lachnoclostridium*, *Faecalicatena*, and *Faecalimonas*. Furthermore, the major species in the high-dose MB intervention group were *Faecalibaculum*, *Lactobacillus*, *Ligilactobacillus*, and *Roseburia_hominis* (Fig. 7G).

### Short-chain fatty acid concentrations

**Fig. 8:**
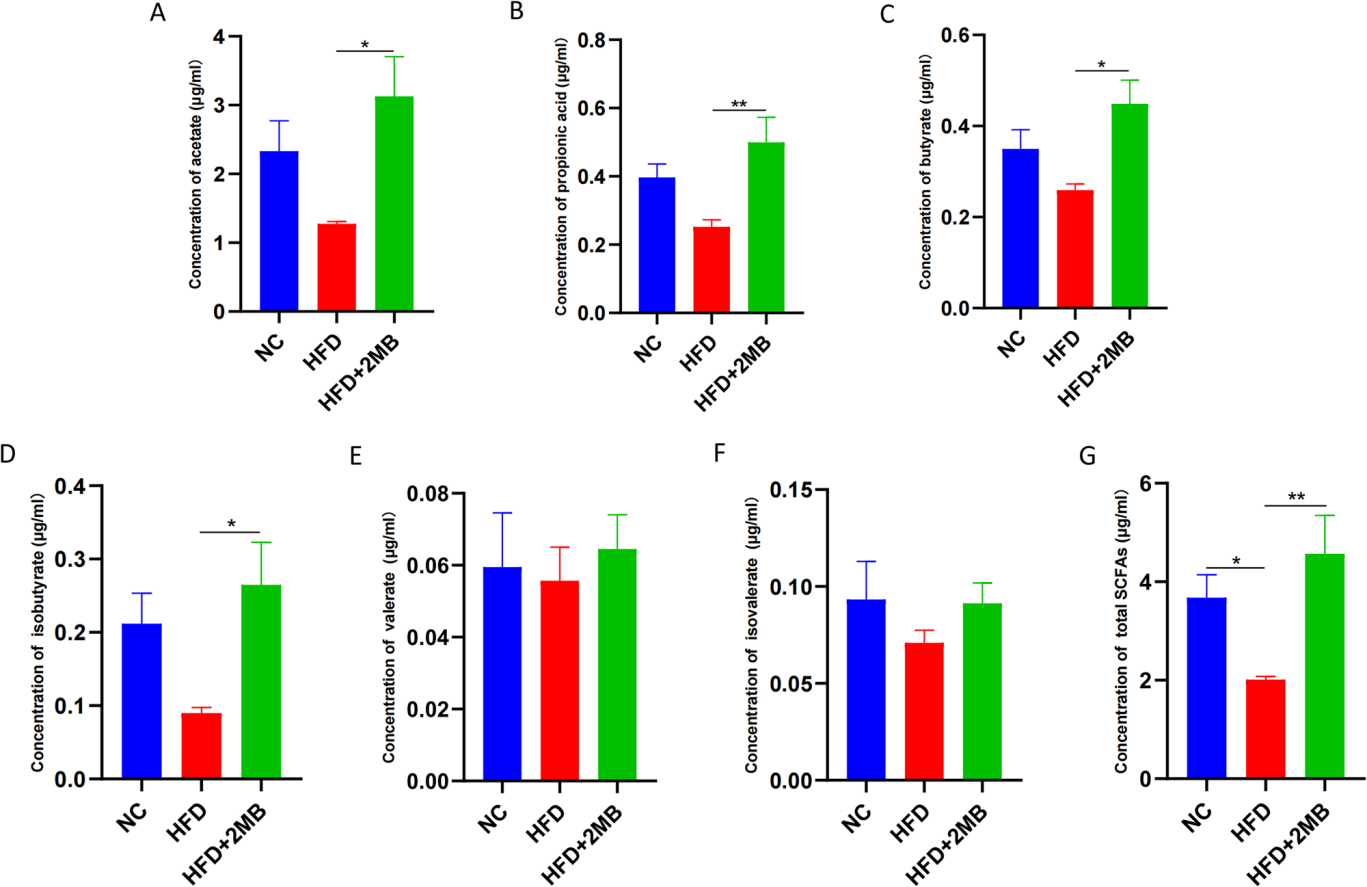
Effect of MB administration on fecal SCFAs level in the colon. **A** The concentration of acetate; **B** The concentration of propanoic acid; **C** The concentration of butyrate; **D** The concentration of isobutyrate; **E** The concentration of valerate; **F** The concentration of isovalerate; **G** The concentration of total SCFAs; Data are presented as mean ± SEM, n = 6. * *p* < 0.05, ** *p* < 0.01.

Total SCFAs concentration in colon contents dramatically decreased in the groups fed a high-fat diet (*p* < 0.05, Fig. 8G). In contrast, the HFD + 2MB group had considerably higher levels of acetic acid, propionic acid, butyric acid, isobutyrate, and total SCFAs than the HFD group (*p* < 0.05, Fig. 8A–G).

## Discussion

The prevalence of NAFLD has increased in parallel with the global epidemic of obesity. At present, the treatment of metabolic diseases such as obesity mainly relies on lifestyle and dietary modifications^18^. In recent years, it has been found that nutrient supplementation has emerged as one of the safest and most effective strategies to improve obesity and metabolic disorders in humans and animals^19^. Previous studies have reported that MB is closely related to the occurrence of metabolic diseases and may play a regulatory role in the pathogenesis of obesity or diabetes^20^. HFD-induced imbalances in lipid metabolism are usually accompanied by body weight gain and fat storage. Similarly, when the weight of the mice was measured after 8 weeks of feeding, our data showed that the HFD-fed mice experienced significant weight loss after receiving MB treatment. As the dose increased, the effect became more prominent. Collectively, the data demonstrated that mice treated with MB could resist diet-induced obesity, and there was no significant difference in feed intake among groups fed high-fat diets, suggesting that the effect of MB on alleviating obesity was not related to feed intake.

When lipid synthesis and degradation in the body are imbalanced, excessive fat accumulates in the liver, resulting in hepatomegaly^21^. Therefore, in this experiment, HFD increased liver weight in obese mice, while MB treatment reduced it. In addition, we found that long-term feeding of mice with HFD resulted in significant hepatic lipid accumulation. The MB intervention significantly alleviated the elevated levels of TC in the serum caused by HFD, which was coincident with an earlier study. Nguyen et al. found that rats fed with monobutyrin exhibited a dose-dependent decrease in total cholesterol and triglycerides in the liver^20^. It has been reported that high levels of TC and non-esterified fatty acids (NEFA) in the serum contribute to the development of NAFLD and other metabolic diseases^22^. In parallel, through histological analysis and Oil Red O staining, it was found that a high-fat diet caused vacuolization of liver tissue and the accumulation of a large number of lipid droplets, indicating the high-fat diet-induced hepatic steatosis in mice. However, MB intake significantly alleviated the liver histological alterations, including extensive hepatocellular vacuolization and lipid droplet accumulation induced by HFD, and the above results preliminarily indicated that MB could alleviate hepatic steatosis in obese mice. AST and ALT are two key aminotransferases in the body. When liver function is impaired, ALT and AST from the cytoplasm are released into the circulatory system^23^. Excessive hepatic fat accumulation can damage liver function, as evidenced by elevated serum AST and ALT levels in this study. MB supplementation significantly reduced these enzyme levels in HFD-fed mice, indicating a protective effect of MB on liver injury.

The AMPK pathway is a classic pathway that regulates lipid metabolism and plays a key role in regulating the balance between cellular catabolism and synthesis metabolism^24^. Activated AMPK has been found to ameliorate NAFLD by increasing hepatic fatty acid oxidation. Additionally, AMPK can upregulate the expression of PPARα^25^. Fatty acid oxidation mainly occurs in mitochondria, and PPARα is an important regulatory factor for hepatic mitochondrial β oxidation^26^. ATGL and HSL are essential rate-limiting enzymes in lipolysis, and the oxidation of long-chain fatty acids initially takes place in peroxisomes before being further degraded in the mitochondria^27^. Furthermore, CPT1, located on the outer mitochondrial membrane, regulates the transport of fatty acids into mitochondria for oxidation^28^. PPARα activation promotes liver fatty acid oxidation by regulating the transcription of related genes, including CPT1 and Acyl-CoA oxidase (ACOX)^29^. In contrast, PPARγ and FAS are associated with the synthesis of fatty acids and contribute to the risk of fatty liver disease. SREBP-1c is a transcriptional activator of fatty acid biosynthesis and induces hepatic steatosis by increasing TG accumulation^30^. The qPCR results indicated that MB treatment upregulated the expression of fatty acid oxidation-related genes (CPT1, PPARα), lipolysis-related genes (ATGL), and energy metabolism regulators such as AMPK, while downregulating fatty acid synthesis genes PPARγ and FAS in the liver. Taken together, our findings indicated that MB supplementation effectively reduced lipid deposition in hepatic tissue by enhancing fatty acid transport and oxidation.

In the current study, RNA-seq results demonstrated that MB treatment up-regulated the expression of PPARα, Acox2, Cpt1, Cpt2, and Fabp2, which were involved in lipid fatty acid oxidation and consistent with the results of mRNA expression of the above potential molecular mechanism of MB in modulating hepatic lipid metabolism. PPARα is primarily expressed in hepatic cells and plays a crucial role in fatty acid β oxidation, the key process of lipid catabolism in the liver. Notably, PPARα agonists were found to ameliorate liver injury by increasing antioxidant enzyme activity, reducing oxidative stress and apoptosis, and decreasing energy metabolism^31^. In the present study, MB activated the expression of PPARα, Acox2, and Cpt1, thereby promoting fatty acid utilization, which may partially explain the decreased TC level in the livers of HFD-fed mice. However, after MB treatment, the expression of PPARγ, Fas, Acsf3, Acsl4, Elovl3, and Elovl5, which are involved in fatty acid synthesis, thus reducing lipid accumulation. ACSL4 is highly selective for free fatty acid synthesis and can preferentially catalyze the reaction between long-chain polyunsaturated fatty acids and coenzyme A. ACSL4-mediated long-chain fatty acid metabolism has been identified as a critical signal for hepatic ferroptosis^32^. When Elovl2 activity is impaired, endoplasmic reticulum stress increases, leading to mitochondrial dysfunction and interfering with lipid production^33^. Moreover, genes associated with fatty acid transport, such as CD36, Apob, and Fabp2 were altered by HFD feeding, whereas these alterations were reverted after MB treatment. Several transporter proteins, including the scavenger receptor CD36 and FABP, are known to mediate cellular fatty acid uptake and contribute to intracellular lipid homeostasis^34^. Similarly, apolipoprotein B (APOB) is a component of numerous lipoprotein particles and serves as a ligand for membrane receptors involved in lipoprotein uptake^35^. Altogether, these results suggested that the administration of MB could improve lipid homeostasis in the hepatic tissues of HFD-fed mice, mainly by mediating the PPARα signaling pathway.

Oxidative stress is the critical element in the pathogenesis of NAFLD, with lipid peroxidation thought to be the primary cause of the “second strike”^36^. Long-term exposure to high cholesterol levels disrupts the equilibrium of lipid metabolism in liver cells, resulting in lipid degeneration and excessive ROS generation due to lipid toxicity^37^. ROS-induced depolarization of mitochondrial membrane potential is a key trigger for mitochondrial autophagy^38^. The liver, a vital metabolic organ, is responsible for free radical scavenging and maintaining lipid homeostasis. MDA is a key indicator of oxidative stress, reflecting the degree of lipid peroxidation. In addition, changes in antioxidant enzyme levels serve as significant indicators of oxidative stress^39^. The physiological role of glutathione peroxidase (GSH-Px) is to decompose reduced GSH and hydrogen peroxide, thereby limiting lipid peroxidation^40^. SOD is essential for scavenging free radicals, thus protecting cells from oxidative damage^41^. CAT, as an oxidoreductase, is mainly found in peroxisomes and can rapidly decompose hydrogen peroxide, a toxic substance produced during cellular metabolism. It has been found that CAT activity is associated with NAFLD, and the knockdown of CAT leads to increased hepatic lipid accumulation^42^. The results showed that MB enhanced the activity of the antioxidant enzyme GSH-Px and reduced hepatic levels of MDA and ROS, thereby increasing hepatic antioxidant capacity and mitigating damage caused by oxidative stress. Indeed, the role of the Nrf2 signaling pathway in liver disease, especially in terms of oxidative stress, has been widely studied. Nrf2 is normally sequestered in the cytoplasm by Keap1, where its basal level is maintained through proteasomal degradation. Upon activation, Nrf2 translocates to the nucleus and promotes the transcription of antioxidant genes such as HO-1 and NQO1, which work together to eliminate excess free radicals and alleviate oxidative stress^43^. Meanwhile, under stress, the liver develops protein folding abnormalities and disruptions in endoplasmic reticulum homeostasis. ER stress also occurs simultaneously with oxidative stress, mitochondrial dysfunction, and impaired autophagy^44^. GRP78 is a molecular chaperone localized in the ER that senses ER stress and facilitates proper protein folding. The activation of these sensors promotes the restoration of endoplasmic reticulum homeostasis by regulating multiple downstream signaling molecules such as ATF4 and CHOP^45^. In this study, we confirmed that MB alleviates ER stress by downregulating the gene expression of hepatic ER stress markers such as CHOP, and by upregulating the expression of HO-1, Nrf2, and Keap1. The comprehensive results indicate that MB may activate the Nrf2 signaling pathway and enhance the levels of antioxidant enzymes, consequently reducing the oxidative stress and relieving endoplasmic reticulum stress in the liver of HFD-fed mice.

Most current studies on humans and mice have shown that the impairment of autophagy in the liver is closely related to the occurrence of fatty liver. Moreover, mitochondrial autophagy in liver cells can enhance the degradation of excess accumulated lipids, which helps to reduce lipid toxicity and the damage caused by oxidative and ER stress^46^. AMPK/mTOR is a primary signaling pathway involved in hepatic cell lipophagy. Various traditional Chinese medicine extracts have been reported to activate AMPK, inhibit downstream mTOR expression, increase the expression of autophagy-related proteins LC3 and p62, and accelerate lipid metabolism in hepatic cells^47^. LC3 microtubule-associated protein is a marker protein located on the autophagosome membrane, while p62, an autophagy-specific substrate, binds to LC3 to recruit ubiquitinated proteins into autophagosomes. Normal autophagic flux is typically indicated by an increase in LC3-II levels and a decrease in p62 levels^48^. Furthermore, studies have shown that the loss of Beclin 1, an essential autophagy regulator, severely disrupts mitochondrial autophagy in mice and significantly reduces fat breakdown^49^. Upon mitochondrial damage, PINK1 accumulates on the outer mitochondrial membrane and phosphorylates Parkin, thereby activating its E3 ubiquitin ligase activity. The activated Parkin ubiquitinates outer membrane proteins, which are recognized by p62 to recruit LC3 and initiate autophagosome formation for mitophagy^50^. Mao et al. discovered that AMPK/mTOR-mediated autophagy levels were dramatically reduced in obese mice^51^. In parallel, it was reported that P62 and LC3, two important components of autophagosomes, are negatively and positively linked with autophagy flux, respectively, and are involved in AMPK-mediated lipophagy, facilitating the clearance of excessive lipid deposits in liver cells^52^. This study showed that high-fat diet induction up-regulated the expression of LC3, Parkin, and Beclin-1 genes, and down-regulated the expression of P62 and mTOR, indicating that MB may increase liver mitochondrial autophagy by activating the AMPK/mTOR signaling pathway, which was in line with the results of the above studies. At the same time, the autophagosomes disappeared in the liver ultrastructure of HFD mice, and the mitochondrial cristae were disrupted. However, the liver mitochondrial structure of the MB group was clear, and there were more autophagosomes capable of eliminating damaged mitochondria, which also confirmed that MB could stabilize the mitochondrial ultrastructure and promote autophagy in the liver.

Previous studies have shown that dysbiosis of gut microbiota in obesity contributes to hepatic oxidative stress by promoting endotoxin translocation, inflammatory responses, and reactive oxygen species (ROS) production^53^. Additionally, altered gut microbiota and reduced SCFA levels can impair mitochondrial function and autophagic processes in hepatocytes^54^. In our study, based on the 16S rRNA analysis of normal mice and obesity mice feces, Chao1 and the Shannon diversity index decreased in the HFD group, indicating lower bacterial diversity in obese mice, but MB intervention seemed to alleviate adverse effects. Here, we observed a significant elevation in the proportion of *Firmicutes* in HFD-fed mice, which was in agreement with many previous studies^55^. Certain components of Firmicutes may translocate into the circulation through the damaged intestinal barrier, triggering metabolic endotoxemia, insulin resistance, and fat accumulation^56^. When treated with MB, the richness of *Lactobacillus*, *Ligilactobacillus*, and *Roseburia_hominis* was significantly increased. *Ligilactobacillus* and *Roseburia_hominis* are widely regarded as the beneficial genera with anti-obesity properties because of their SCFAs-producing ability, which break down indigestible carbohydrates and preserve intestinal barrier function^57,58^. In particular, *Lactobacillus* is probiotic bacterium used as dietary supplement that inhibits the synthesis of endogenous cholesterol, which may be related to ameliorating the disturbance of lipid metabolism^59^. Among them, *Lachnoclostridium* was found to be more abundant in HFD-induced obese rats and was associated with reduced acetate levels, potentially contributing to metabolic dysregulation^60^. *Faecalimonas* is a bacterium that produces skatole, which is linked to chronic inflammation as observed in cases of obesity^61^. In this work, these results suggest that MB intervention may alleviate gut microbiota dysbiosis induced by HFD and improve intestinal health by enriching SCFA-producing beneficial bacteria. Modulation of the gut-liver axis may represent an important mechanism by which MB exerts its hepatoprotective effects. However, further mechanistic studies are needed to confirm this interplay and establish causality.

To sum up, our findings indicated that MB ameliorated the lipid metabolism issue and inhibited hepatic lipogenesis by regulating the PPARα signaling pathway to promote fatty acid oxidation. MB treatment also reduced HFD-induced oxidative stress injury in the liver by enhancing peroxidase activity, maintaining mitochondrial ultrastructure, and promoting autophagy. Meanwhile, MB alleviated HFD-induced obesity by modulating gut microbiota, which enhanced short-chain fatty acids synthesis. These results provide a novel perspective on the therapeutic role of MB in NAFLD.

## Methods

### Preparation of MB

MB was synthesized by J&K Scientific (90% purity, Beijing, China). The MB solution used for intragastrical administration was prepared daily in phosphate-buffered saline (PBS), and the solution underwent ultrasonic emulsification for 30 min in an ultrasonic cleaner at room temperature.

### Mice and experimental design

**Fig. 9:**
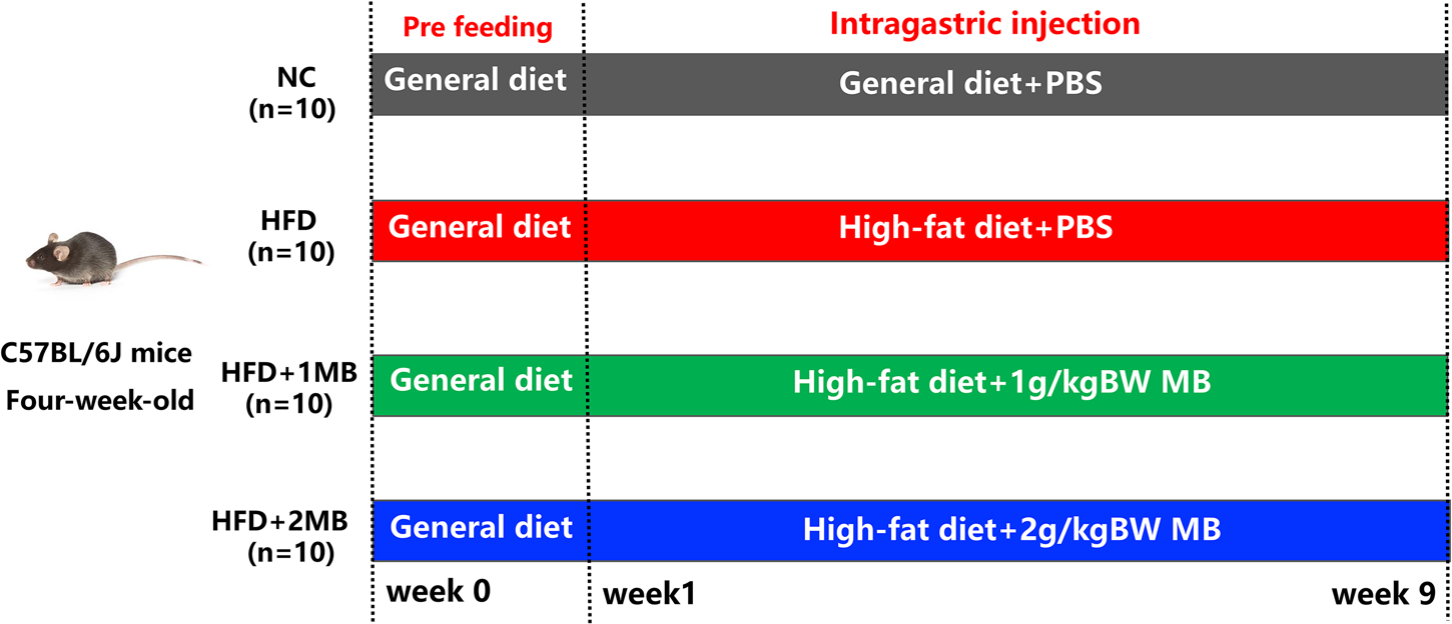
Schematic for the feeding experiment. Male C57BL/6J mice received the general or high-fat diet for nine weeks to establish the model. Then, the mice were treated with phosphate buffer saline (PBS), 1 g/kg BW MB, or 2 g/kg BW by gavage in 0.2 mL volumes for eight weeks.

A total of 40 healthy four-week-old C57BL/6 male mice were purchased from the Zhejiang Academy of Medical Sciences (Hangzhou, China) and were fed at the Zhejiang University Laboratory Animal Center. The experimental design is shown in Fig. 9. All mice were housed in cages maintained at a 12 h light and dark cycle at 25 °C with ad libitum access to distilled water. After one week of adaptive feeding, all mice were randomly assigned to 4 groups as follows: (1) NC group (n = 10): mice were fed a basal diet (including 70% energy from carbohydrate, 20% energy from protein, and 10% energy from fat, 3601 kcal/kg) and oral gavage with 0.2 mL PBS per day; (2) HFD group (n = 10): mice were fed a high-fat diet (including 60% energy from carbohydrate, 20% energy from protein, and 20% energy from fat, 5128 kcal/kg) and oral gavage with 0.2 mL PBS per day; (3) HFD + 1MB group (n = 10): mice were fed a high-fat diet and oral gavage with MB (1 g/kg BW·day); (4) HFD + 2MB group (n = 10): mice were fed a high-fat diet and oral gavage with MB (2 g/kg BW·day). The MB dosages (1 and 2 g/kg BW) were selected based on our earlier study and literature^20,62,63^. The nutritional composition of the basal diet and the high-fat diet used throughout the study is shown in Table 4. The body weights and energy intake per mouse were collected weekly. After diet intervention for 8 weeks, blood samples were collected from the retro-orbital plexuses, then centrifuged at 3000 × *g* for 10 min to isolate serum, and all mice were immediately euthanized by cervical dislocation. The liver was removed, weighed, and subsequently collected in sterile centrifuge tubes. All the samples were snap-frozen in liquid nitrogen and stored at −80 °C for further analysis. The study was conducted according to the ethical guidelines of Zhejiang University’s Institutional Animal Care and Use Committee approved and governed all experimental protocols and animal care (approval number: ZJU20250085).

**Table 4:** The nutrient class and ingredients of the diets

Nutrient class	Ingredient	Basal diet (g)	High fat diet (g)
Protein	Casein	200	200
	L-Cystine	3	3
Carbohydrate	Corn Starch	506.2	0
	Maltodextrin 10	125	125
	Sucrose	72.8	72.8
Fiber	Celulose	50	50
Fat	Soybean oil	25	25
	Lard	20	245
Mineral	Mineral Mix S10026B	50	50
Vitamin	Vitamin Mix V10001C	1	1
	Choline Bitartrate	2	2

### Biochemical index and antioxidant capacity measurement

The activities of serum alanine aminotransferase (ALT) and aspartate aminotransferase (AST) were measured using the automatic biochemical analyzer (Olympus AU2700, Tokyo, Japan) at Affiliated Hospital of Hangzhou Normal University (Hangzhou, China). The contents of total cholesterol (TC) (A111-1-1), total triglyceride (TG) (A110-1-1), and the levels of glutathione peroxidase (GSH-Px) (A005-1), catalase (CAT) (A007-1), superoxide dismutase (SOD) (A001-3) and malondialdehyde (MDA) (A003-1) in liver were quantified using the corresponding commercial kits (Nanjing Jiancheng Bioengineering Institute, China) according to the manufacturer’s instructions.

### Reactive oxygen species (ROS) determinations

Fresh liver tissues of mice were immediately sampled and stored at −20 °C, after which they were made into 10 µm frozen sections. Besides, a part of OCT-frozen liver tissue was used to evaluate the oxidative stress via ROS assay kit by using dihydroethidium (DHE) as the fluorescent probe (D7008-10, Sigma-Aldrich, USA). Furthermore, the fluorescence images were obtained via fluorescence microscopy (Olympus, BX53, Japan).

### Liver histological assessment

The portion of fresh liver was excised and fixed in 4% paraformaldehyde (PFA) for 24 h. After fixing, the tissues were dehydrated with ethanol and embedded in paraffin. Tissue sections (5 μm) were cut and stained with hematoxylin-eosin (H&E) staining to observe the pathological changes. Additionally, frozen sections of 5 μm liver were stained with Oil Red O to visualize the distribution of lipid droplets. Observe and capture images under an optical microscope (Nikon Eclipse 80i, Tokyo, Japan).

### Liver ultrastructural observation

The fresh liver tissue for transmission electron microscope (TEM) analysis was fixed in 2.5% glutaraldehyde-PBS solution for 48 h. After rinsing phosphate-buffered saline, the specimens were further immersed in 1% osmium tetroxide for 1 h. Following ethanol dehydration and embedding, the samples were cut at a thickness of 70–90 nm. Then, these sections were counterstained with Mg-uranyl acetate and Pb-citrate, and the ultrastructure was observed under a transmission electron microscope (Hitachi HT 7650, Tokyo, Japan).

### Real-time quantitative PCR

The RNA was extracted from the liver using the TRIzol reagent (Invitrogen, Carlsbad, CA, USA). RNA quantity was measured using the NanoDrop 2000 spectrophotometer (Thermo Fisher Scientific, Waltham, MA, USA). Then, total RNA was reverse-transcribed to cDNA using the PrimeScrip RT reagent Kit with gDNA Eraser Perfect Real-Time (Takara Biomedical Technology, Beijing, China), and the gene expression levels were determined using a CFX96™ qPCR system (Bio-Rad, Hercules, CA, USA) according to the product protocols. Primer sequences of the β-actin, carnitine palmitoyltransferase1 (*CPT1*), peroxisome proliferator-activated receptors α (*PPARα*), AMP-activated protein kinase (*AMPK*), Acyl-Coenzyme A oxidase (*ACOX*), peroxisome proliferator-activated receptors γ (*PPARγ*), fatty acid synthase (*FAS*), Sterol-regulatory element binding proteins (*SREBP1*), adipose triglyceride lipase (*ATGL*), hormone-sensitive triglyceride lipase (HSL), heme oxygenase-1 (*HO-1*), nuclear factor erythroid related factor 2 (*Nrf2*), kelch-like epichlorohydrin associated protein-1 (*Keap1*), NADH quinone oxidoreductase 1 (*NQO1*), activating transcription factor 4 (*ATF4*), C/EBP homology protein (*CHOP*), glucose-regulated protein 78 (*GRP78*), mechanistic target of rapamycin kinase (*mTOR*), ubiquitin-binding protein (*P62*), microtubule-associated protein 1A/1B-light chain 3 (*LC3*), PTEN induced putative kinase 1 (*PINK1*), Parkin, autophagy-related protein 5 (*ATG5*), *Beclin1* in the liver are listed in Table 5. All the gene sequences were quoted from NCBI, and the PCR primers were produced in Tsingke (Beijing, China). All the measurements were carried out in triplicate (n = 6), and the average values were calculated. Relative expression levels of different genes were normalized to the expression of β-actin using the 2^−ΔΔCt^ method.

**Table 5:** Primers used in real-time quantitative PCR

Gene	Gene bank ID	Primer sequence (5’-3’)
*β-actin*	NM_007393	F: GGCTGTATTCCCCTCCATCG
		R: CCAGTTGGTAACAATGCCATGT
*CPT1*	NM_013495.2	F: AGATCAATCGGACCCTAGACAC
		R: CAGCGAGTAGCGCATAGTCA
*PPARα*	NM_001113418.1	F: AGGCTGTAAGGGCTTCTTTC
		R: GCATTTGTTCCGGTTCTTCTTC
*AMPK*	NM_023991.2	F: TGAAGATCGGCC ACTACATCCT
		R: CTTGCCCACCTTCACTTTCC
*ACOX*	NM_001271898.2	F: CGCACATCTTGGATGGTAGT
		R: GGCTTCGAGTGAGGAAGTTATAG
*PPARγ*	NM_001127330.3	F: AGGGCGATCTTGACAGGAAAGAC
		R: AAATTCGGATGGCCACCTCTTTGC
*FAS*	NM_007988.3	F: AGACCCGAACTCCAAGTTATTC
		R: GCAGCTCCTTGTATACTTCTCC
*SREBP1*	NM_001313979.1	F: GCAGCCACCATCTAGCCTG
		R: CAGCAGTGAGTCTGCCTTGAT
*ATGL*	NM_001163689.1	F: CGCGCTCTTGGCTCATG
		R: CCAACCTTTGTGCCCCTTAA
*HSL*	NM_001039507.2	F: CAGGAGAGCAGGGATTTGCA
		R: CCTACGCTCAGCCCTCTTCAT
*HO-1*	NM_010442.2	F: GCTAGCCTGGTGCAAGATACT
		R: AAGCTGAGAGTGAGGACCCA
*Nrf2*	NM_001399226.1	F: ACCTCTGCTGCAAGTAGCCT
		R: TGGGCAACCATCACTCTGCT
*Keap1*	NM_001110305.1	F: GCCCCGGGACTCTTATTGTG
		R: TTAGGGGCCCCGCCAT
*NQO1*	NM_008706.5	F: GTAGCGGCTCCATGTACTCT
		R: AGGATGCCACTCTGAATCGG
*ATF4*	NM_001287180.1	F: AGCCCCACAACATGAC
		R: CCACCTCCAGATAGTCAT
*CHOP*	NM_001290183.2	F: CCACCACACCTGAAAGCAGAA
		R: GGTGCCCCCAATTTCATCT
*GRP78*	NM_001163434.1	F: ACATGGACCTGTTCCGCTCTA
		R: TGGCTCCTTGCCATTGAAGA
*mTOR*	XM_006539077.3	F: CCATCCAATCTGATGCTGGA
		R: GGTGTGGCATGTGGTTCTGT
*P62*	NM_001290769.1	F: TGTGGAACATGGAGGGAAGAG
		R: TGTGCCTGTGCTGGAACTTTC
*LC3*	NM_001364358.1	F: GAGGGGACCCTAACCCCATA
		R: TCGCTCTATAATCACCCGCC
*PINK1*	NM_026880.2	F: GAGGAGCAGACTCCCAGTTC
		R: AGGGACAGCCATCTGAGTCC
*Parkin*	NM_001317726.2	F: GAGGGGAAGGGGGAGGA
		R: GGAGTTGAACCTGACAAACACTAT
*ATG5*	NM_001314013.2	F: ATGGGACTGCAGAATGACAG
		R: CCGGAACAGCTTCTGGATGA
*Beclin1*	NM_001034117.1	F: AACTCTGGAGGTCTCGCTCT
		R: CCTTAGACCCCTCCATTCCTCA

F forward, R reverse.

### Hepatic transcriptome sequencing and bioinformatics analysis

Total liver RNA was extracted using the TRIzol method (Invitrogen, CA, USA) and treated with RNase-free. RNA integrity was assessed using the Agilent 2100 Bioanalyzer (Agilent Technologies, CA, USA). RNA purity and concentration were detected by a Nanodrop spectrophotometer (Thermo Scientific, DE, USA). CDNA library was constructed using NEBNext® Ultra™ RNA Library Prep Kit for Illumina (NEB, USA) following the manufacturer’s recommendations. The library preparations were sequenced on an Illumina Novaseq 6000 platform by the Beijing Allwegene Technology Company Limited (Beijing, China). Raw reads of fastq format were transformed into clean reads by removing the adapter sequences and low-quality sequence reads. These clean reads were then mapped to the reference genome sequence by STAR (V2.5.2 B). Only reads with a perfect match or one mismatch were further analyzed and annotated based on the reference genome. HTSeq software was utilized to analyze the gene expression level of the samples. Differential expression analysis of the three groups was performed using the DESeq (V1.10.1). Genes with an adjusted *P*-value < 0.05 found by DESeq were assigned as differentially expressed. Gene Ontology (GO) enrichment analysis of the differentially expressed genes (DEGs) was implemented by the GOseq (V1.22) software based on the Wallenius non-central hyper-geometric distribution. We used the Kyoto Encyclopedia of Genes and Genomes (KEGG, https://www.kegg.jp/kegg/rest/keggapi.html) to test the statistical enrichment of differentially expressed genes in the KEGG pathway to determine the main biochemical metabolic pathway and signal transduction pathway involved in differentially expressed genes.

### Western blot analysis

The analysis of western blot was performed in line with our previous report^64^, which determined ubiquitin-binding protein (P62) and microtubule-associated protein 1A/1B-light chain 3 (LC3). Protein bands were detected via the ECL chemiluminescence kit (FDbio-Dura, Hangzhou, China). The membrane was exposed by ChemiDoc MP (Bio-Rad Laboratories, America) and the band intensity was detected by ImageJ software. GAPDH was used as the internal reference.

### Immunofluorescence

The paraffin-embedded liver sections underwent a dehydration process using ethanol solutions and were dewaxed in xylene. The sample slides were blocked with 1% goat serum albumin for 1 h at 37 °C, followed by incubation with LC3 (dilution 1:300, GB13431, Servicebio, Wuhan, China), and p62 (dilution 1:500, GB11531, Servicebio, Wuhan, China) antibodies at 4 °C overnight. After that, were further incubated with secondary antibodies for 1 h and cell nuclei were stained with 4,6-diamidino-2-phenylindole (DAPI, G1012, Servicebio, Wuhan, China) for 10 min. Images were obtained using a fluorescence microscope (Olympus). Image J software was used to analyze the images.

### Gut microbiota profiling in feces

Total DNA from colonic content was extracted using a commercial DNA extraction kit (Omega Bio-tek, Inc., USA). The quantity and quality of extracted DNAs were measured using NanoDrop 2000 spectrophotometer (Thermo Scientific Inc., USA). The V3-4 hypervariable region of bacterial 16S rRNA gene was amplified with the universal primer 338 F (5’ -ACTCCTACGGGAGGCAGCAG-3’) and 806 R (5’ -GGACTACNNGGGTATCTAAT-3’). The PCR products were purified using an Agencourt AMPure XP Kit (Beckman Coulter, Inc., USA). Sequencing libraries were generated using NEB Next Ultra II DNA Library Prep Kit (New England Biolabs, Inc., USA), following the manufacturer’s recommendations. Deep sequencing was performed on Illumina Miseq (Illumina, Inc., USA) platform at Beijing Allwegene Technology Co., Ltd. After the run, image analysis, base calling, and error estimation were performed using Illumina Analysis Pipeline Version 2.6 (Illumina, Inc., USA). Sequence data associated with this project have been deposited in the NCBI Short Read Archive database (Accession Number: PRJNA1225918).

### Determination of SCFAs

Targeted metabolomics was performed with GC–MS to quantify the concentrations of SCFAs in colonic content. The contents of the colon were weighed 20 mg, added with 0.1 mL absolute ethyl alcohol, vortexed, and placed at 4 °C for 1 h. After 15 min of centrifugation at 10,000 rpm, 20 µL of 85% metaphosphoric acid solution was added to the 1 mL supernatants, the solution was kept standing for 1 h with an environmental temperature of 4 °C, and then centrifuged at 12,000 × *g* for 15 min at 4 °C. Finally, the supernatants were filtered through 0.22 μm stream filter membrane and transferred into the gas chromatography vial. SCFAs determination using gas chromatography (Agilent 5975C, Santa Clara, CA, USA) and flame ionization detector (FID). The sample size was 1 μL, and the chromatographic column was DB-624 capillary column (30 m × 0.32 mm × 1.8 μm).

### Statistical analysis

Data are presented with mean ± SEM, and all statistical analyses were performed with GraphPad Prism 8.0 software. The normality of the data was tested using the Shapiro-Wilk test, and the significance among the groups was identified using the one-way analysis of variance (ANOVA), followed by Tukey’s post hoc comparison. A *P* value of <0.05 was considered to be statistically significant.

## Supplementary information

Supplementary Information

## Data Availability

The authors declare that the data supporting the findings of this study are available within the paper. Any raw data files in another format used to support the findings of this study are available from the corresponding author upon reasonable request.
