# Supplementary material for: Monobutyrin can alleviate hepatic lipid dysmetabolism and improve liver mitochondrial ultrastructure and autophagy in high-fat diet mice
Source: NPJ Sci Food. 2025 Jul 29;9:159. doi: 10.1038/s41538-025-00524-6 (PMC12307903; doi:10.1038/s41538-025-00524-6)

Uncropped and unprocessed scans of blots for Figure 5B.

Original western blot of LC3 and P62 for three repeats.

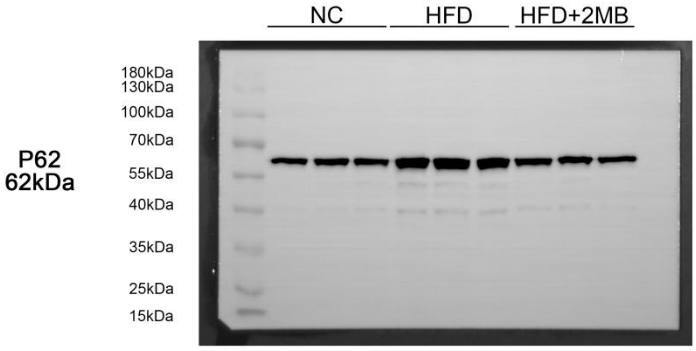

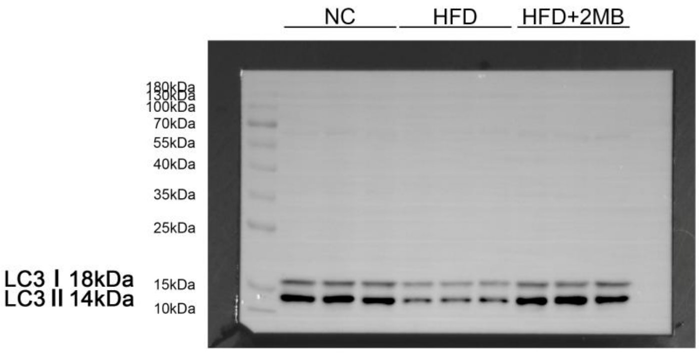

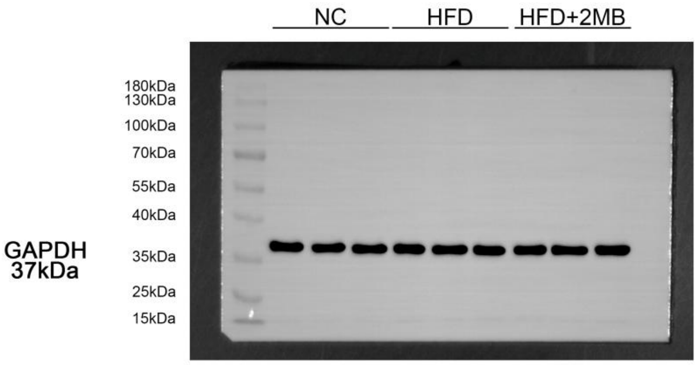

Supplement: Supplementary file 1 — Supplementary Information [file 41538_2025_524_MOESM1_ESM.pdf]
